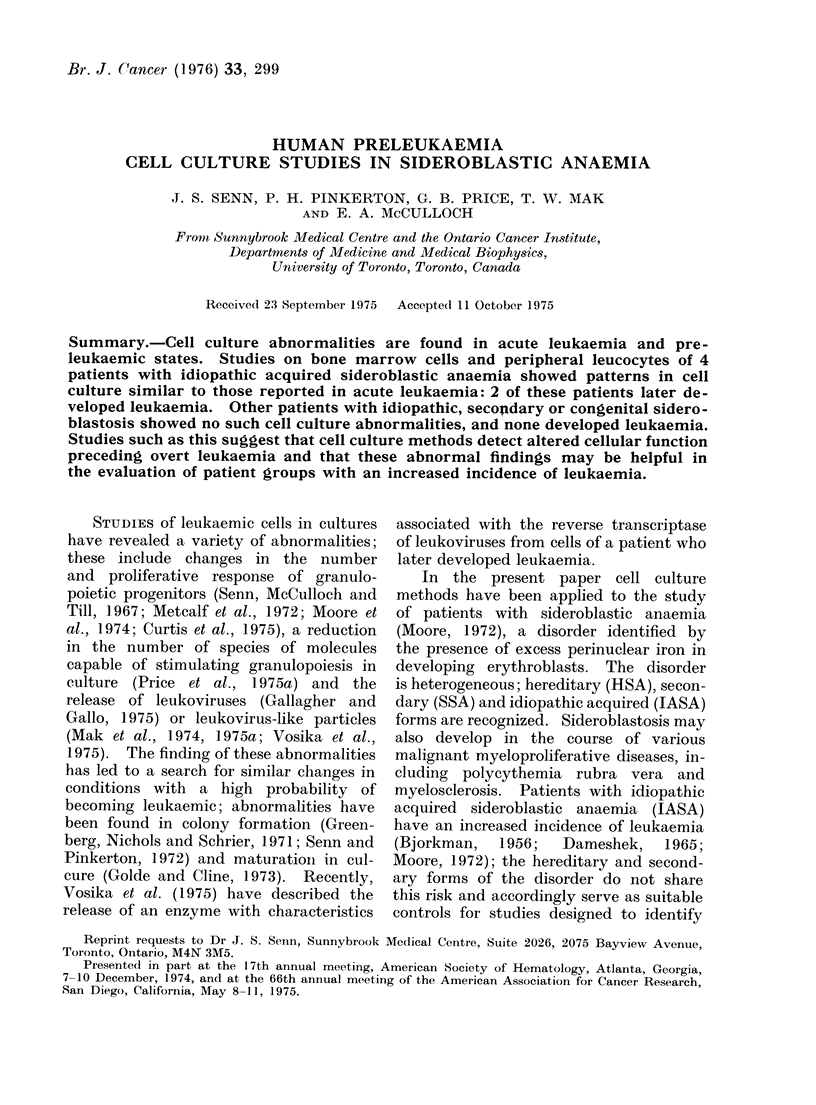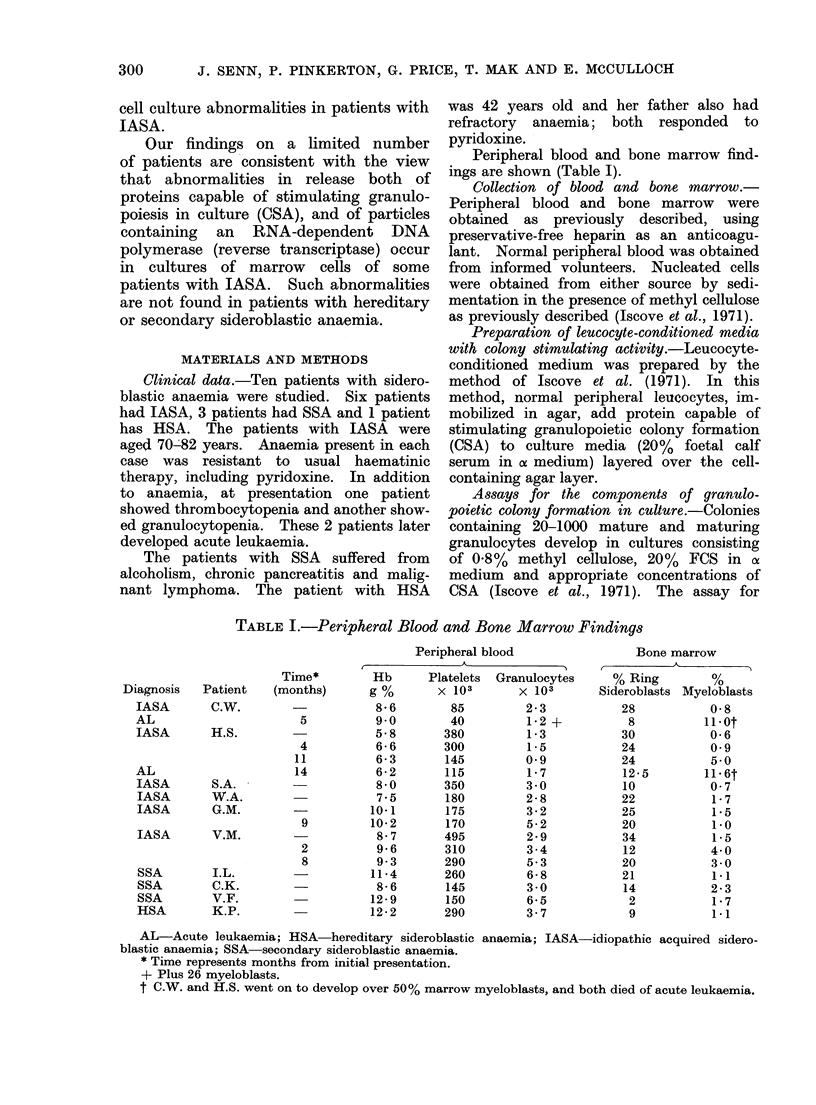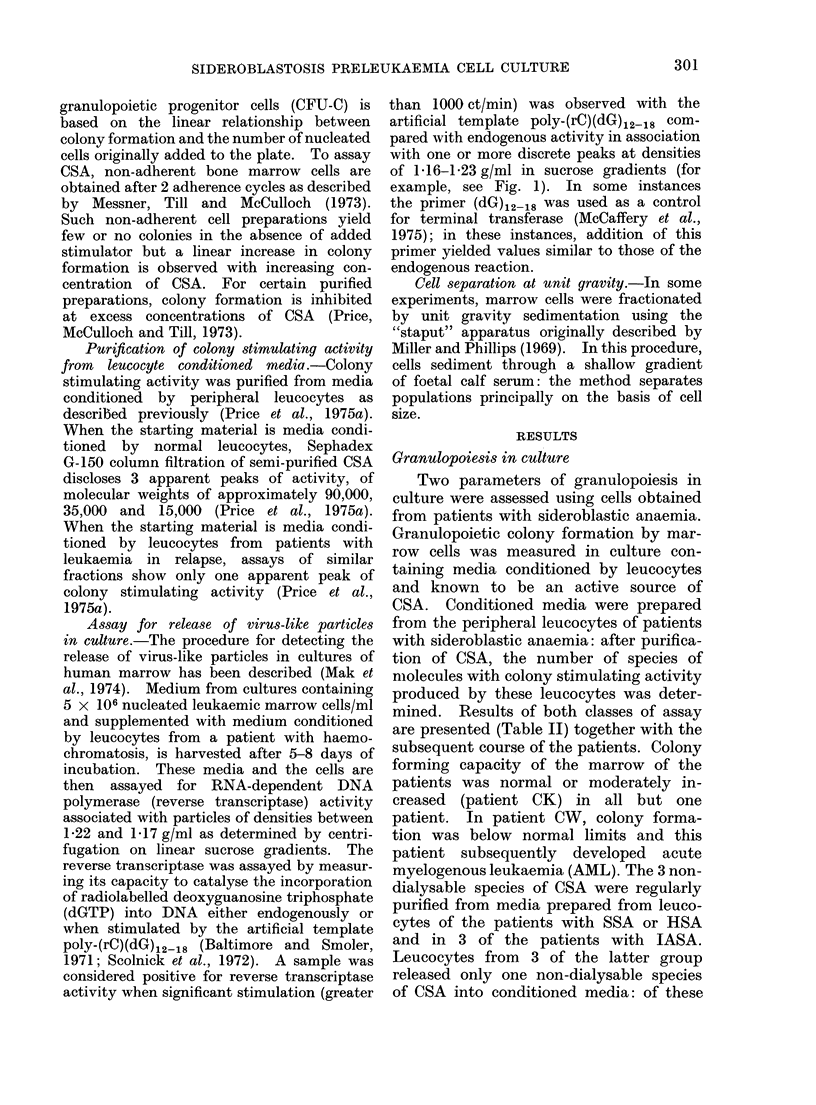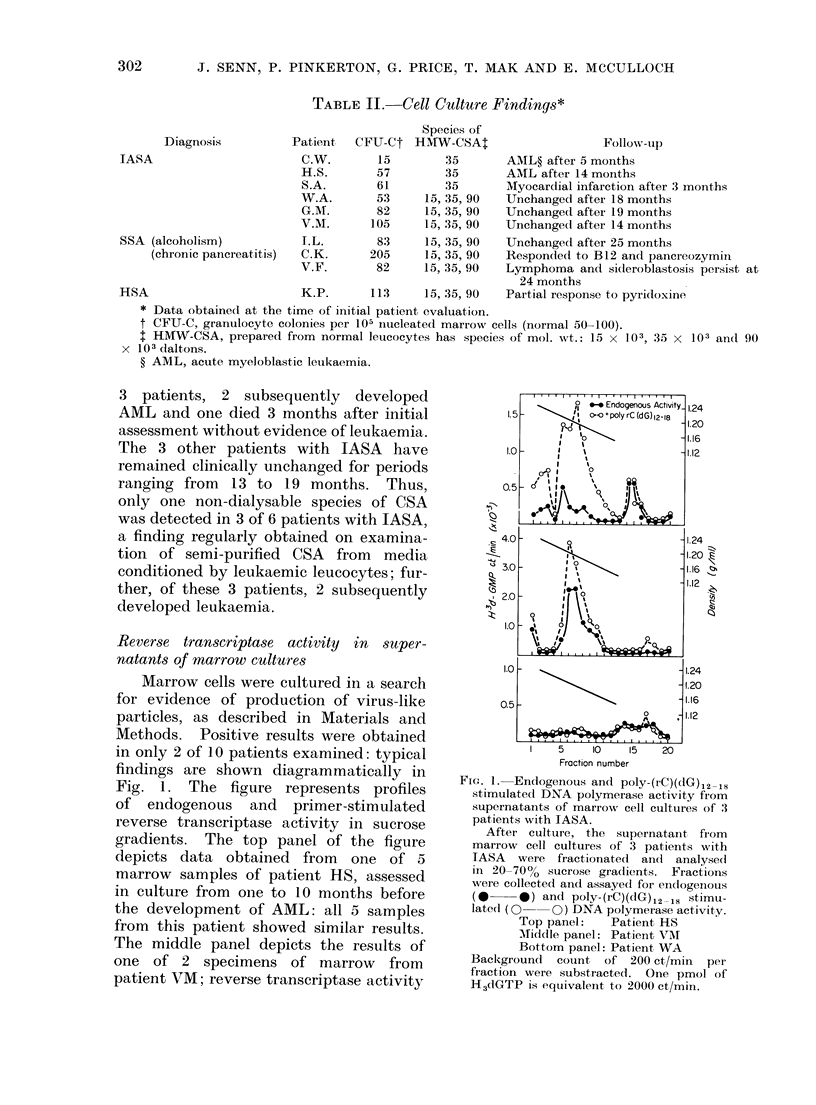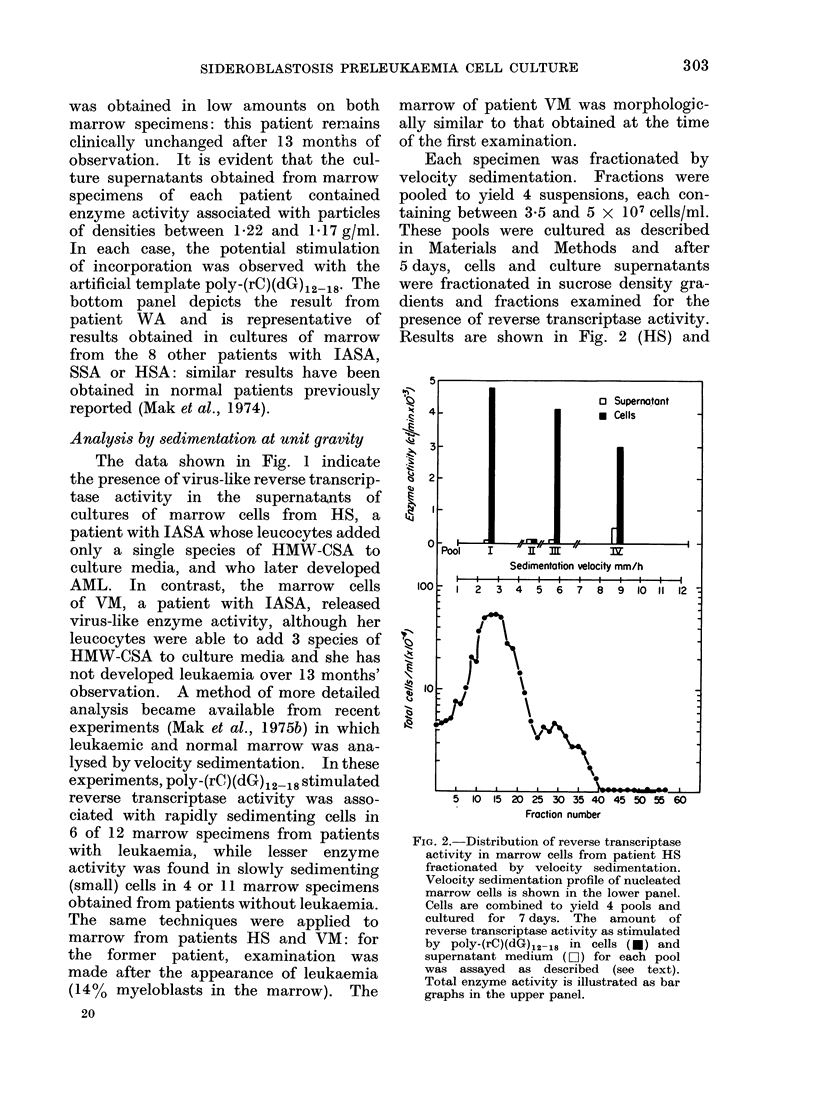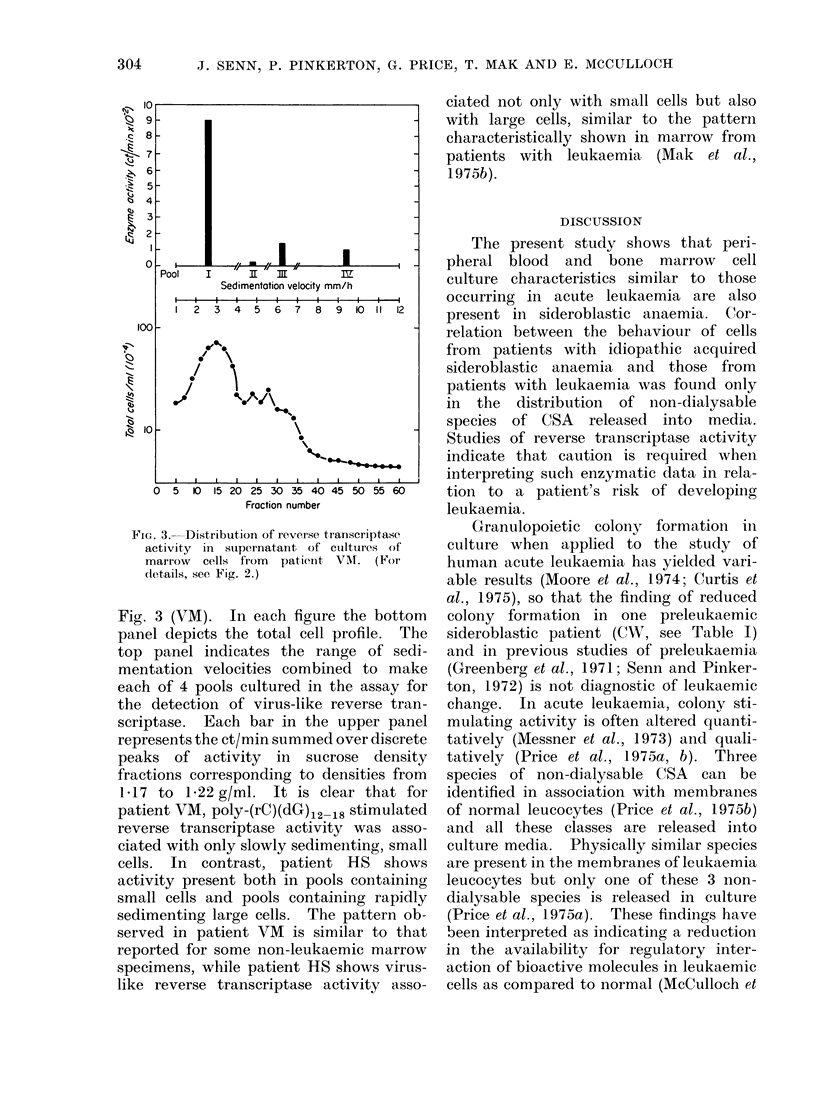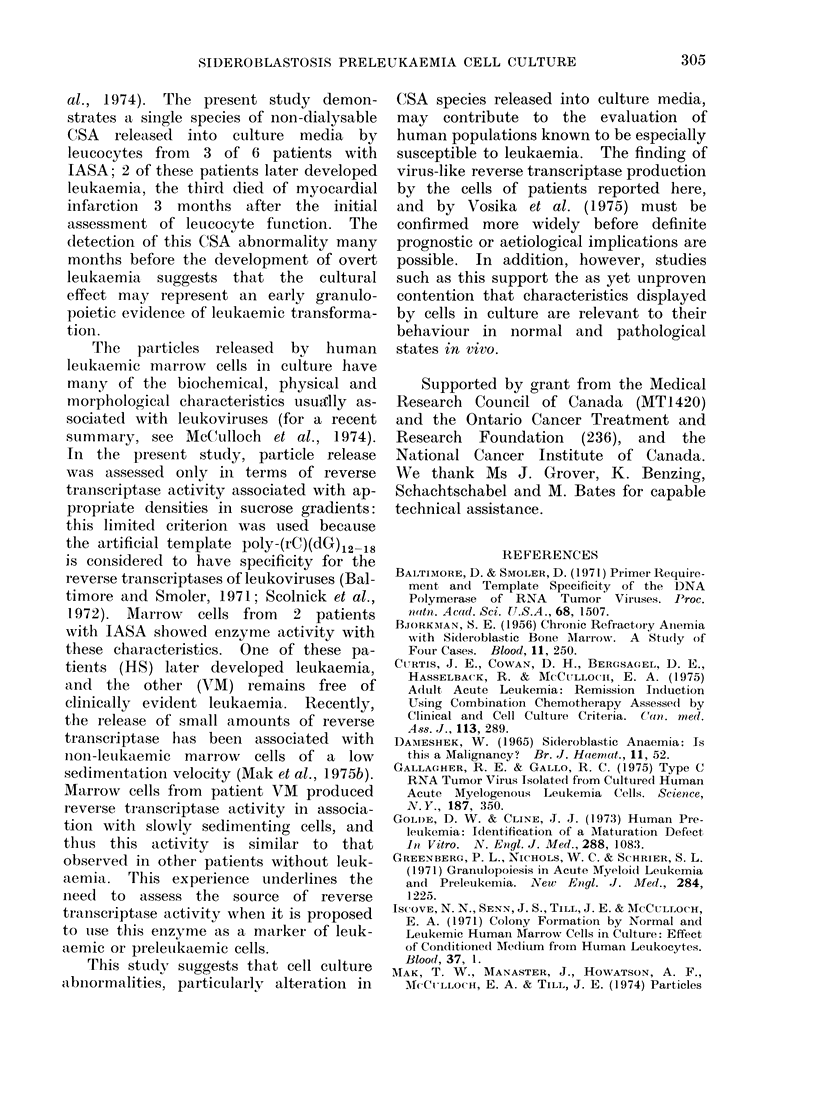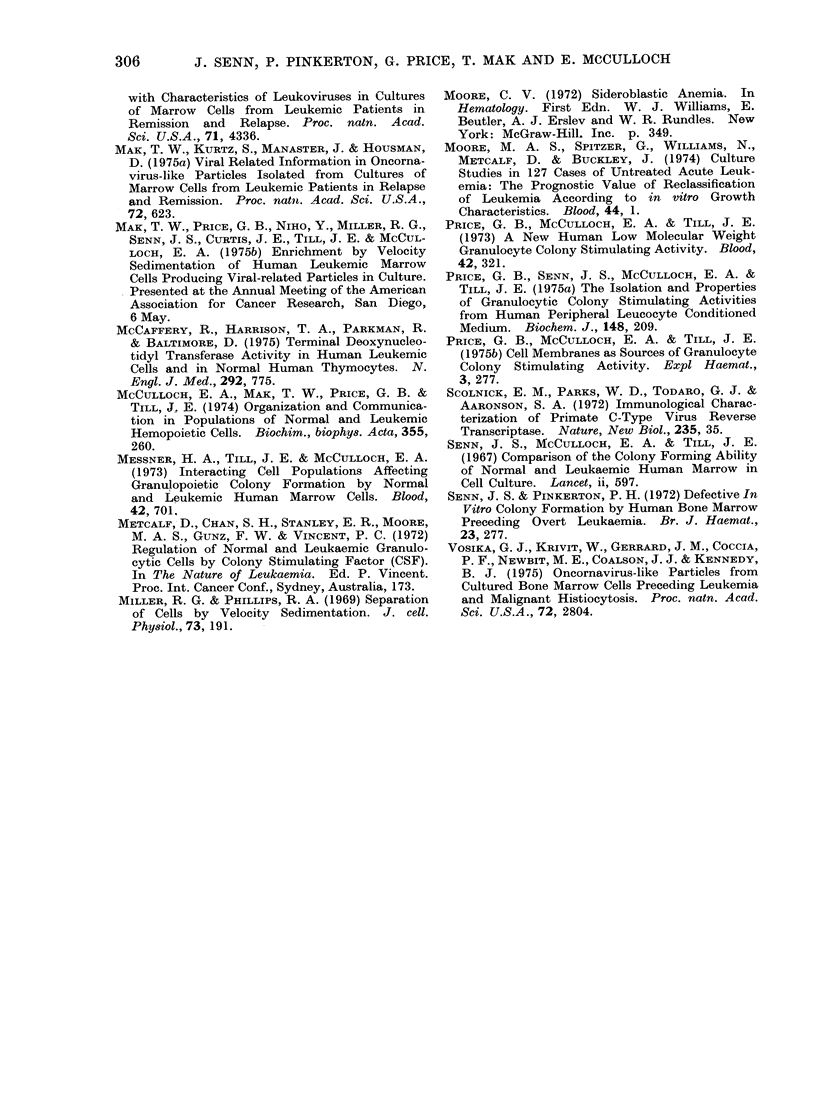# Human preleukaemia cell culture studies in sideroblastic anaemia.

**DOI:** 10.1038/bjc.1976.43

**Published:** 1976-03

**Authors:** J. S. Senn, P. H. Pinkerton, G. B. Price, T. W. Mak, E. A. McCulloch

## Abstract

Cell structure abnormalties are found in acute leukaemia and preleukaemic states. Studies on bone marrow cells and peripheral leucocytes of 4 patients with idiopathic acquired sideroblastic anaemia showed patterns in cell culture similar to those reported in acute leukaemia: 2 of these patients later developed leukaemia. Other patients with idiopathic, secondary or congenital sideroblastosis showed no such cell culture abnormalities, and none developed leukaemia. Studies such as this suggest that cell culture methods detect altered cellular function preceding overt leukaemia and that these abnormal findings may be helpful in the evaluation of patient groups with an increased incidence of leukaemia.


Br. J. Cancer (1976) **33**, 299

### HUMAN PRELEUKAEMIA
### CELL CULTURE STUDIES IN SIDEROBLASTIC ANAEMIA

J. S. SENN, P. H. PINKERTON, G. B. PRICE, T. W. MAK AND E. A. McCULLOCH

*From Sunnybrook Medical Centre and the Ontario Cancer Institute, Departments of Medicine and Medical Biophysics, University of Toronto, Toronto, Canada*

Received 23 September 1975 Accepted 11 October 1975

**Summary.—Cell culture abnormalities are found in acute leukaemia and preleukaemic states. Studies on bone marrow cells and peripheral leucocytes of 4 patients with idiopathic acquired sideroblastic anaemia showed patterns in cell culture similar to those reported in acute leukaemia: 2 of these patients later developed leukaemia. Other patients with idiopathic, secondary or congenital sideroblastosis showed no such cell culture abnormalities, and none developed leukaemia. Studies such as this suggest that cell culture methods detect altered cellular function preceding overt leukaemia and that these abnormal findings may be helpful in the evaluation of patient groups with an increased incidence of leukaemia.**

STUDIES of leukaemic cells in cultures have revealed a variety of abnormalities; these include changes in the number and proliferative response of granulopoietic progenitors (Senn, McCulloch and Till, 1967; Metcalf *et al.*, 1972; Moore *et al.*, 1974; Curtis *et al.*, 1975), a reduction in the number of species of molecules capable of stimulating granulopoiesis in culture (Price *et al.*, 1975*a*) and the release of leukoviruses (Gallagher and Gallo, 1975) or leukovirus-like particles (Mak *et al.*, 1974, 1975*a*; Vosika *et al.*, 1975). The finding of these abnormalities has led to a search for similar changes in conditions with a high probability of becoming leukaemic; abnormalities have been found in colony formation (Greenberg, Nichols and Schrier, 1971; Senn and Pinkerton, 1972) and maturation in culcure (Golde and Cline, 1973). Recently, Vosika *et al.* (1975) have described the release of an enzyme with characteristics associated with the reverse transcriptase of leukoviruses from cells of a patient who later developed leukaemia.

In the present paper cell culture methods have been applied to the study of patients with sideroblastic anaemia (Moore, 1972), a disorder identified by the presence of excess perinuclear iron in developing erythroblasts. The disorder is heterogeneous; hereditary (HSA), secondary (SSA) and idiopathic acquired (IASA) forms are recognized. Sideroblastosis may also develop in the course of various malignant myeloproliferative diseases, including polycythemia rubra vera and myelosclerosis. Patients with idiopathic acquired sideroblastic anaemia (IASA) have an increased incidence of leukaemia (Bjorkman, 1956; Dameshek, 1965; Moore, 1972); the hereditary and secondary forms of the disorder do not share this risk and accordingly serve as suitable controls for studies designed to identify

Reprint requests to Dr J. S. Senn, Sunnybrook Medical Centre, Suite 2026, 2075 Bayview Avenue, Toronto, Ontario, M4N 3M5.

Presented in part at the 17th annual meeting, American Society of Hematology, Atlanta, Georgia, 7–10 December, 1974, and at the 66th annual meeting of the American Association for Cancer Research, San Diego, California, May 8–11, 1975.

cell culture abnormalities in patients with IASA.

Our findings on a limited number of patients are consistent with the view that abnormalities in release both of proteins capable of stimulating granulopoiesis in culture (CSA), and of particles containing an RNA-dependent DNA polymerase (reverse transcriptase) occur in cultures of marrow cells of some patients with IASA. Such abnormalities are not found in patients with hereditary or secondary sideroblastic anaemia.

### MATERIALS AND METHODS

*Clinical data.*—Ten patients with sideroblastic anaemia were studied. Six patients had IASA, 3 patients had SSA and 1 patient has HSA. The patients with IASA were aged 70–82 years. Anaemia present in each case was resistant to usual haematinic therapy, including pyridoxine. In addition to anaemia, at presentation one patient showed thrombocytopenia and another showed granulocytopenia. These 2 patients later developed acute leukaemia.

The patients with SSA suffered from alcoholism, chronic pancreatitis and malignant lymphoma. The patient with HSA was 42 years old and her father also had refractory anaemia; both responded to pyridoxine.

Peripheral blood and bone marrow findings are shown (Table I).

*Collection of blood and bone marrow.*—Peripheral blood and bone marrow were obtained as previously described, using preservative-free heparin as an anticoagulant. Normal peripheral blood was obtained from informed volunteers. Nucleated cells were obtained from either source by sedimentation in the presence of methyl cellulose as previously described (Iscove *et al.*, 1971).

*Preparation of leucocyte-conditioned media with colony stimulating activity.*—Leucocyte-conditioned medium was prepared by the method of Iscove *et al.* (1971). In this method, normal peripheral leucocytes, immobilized in agar, add protein capable of stimulating granulopoietic colony formation (CSA) to culture media (20% foetal calf serum in α medium) layered over the cell-containing agar layer.

*Assays for the components of granulopoietic colony formation in culture.*—Colonies containing 20–1000 mature and maturing granulocytes develop in cultures consisting of 0·8% methyl cellulose, 20% FCS in α medium and appropriate concentrations of CSA (Iscove *et al.*, 1971). The assay for

TABLE I.—*Peripheral Blood and Bone Marrow Findings*

| | | | Peripheral blood | | | Bone marrow | |
|---|---|---|---|---|---|---|---|
| Diagnosis | Patient | Time* (months) | Hb g % | Platelets × 10³ | Granulocytes × 10³ | % Ring Sideroblasts | % Myeloblasts |
| IASA | C.W. | — | 8·6 | 85 | 2·3 | 28 | 0·8 |
| AL | | 5 | 9·0 | 40 | 1·2 + | 8 | 11·0† |
| IASA | H.S. | — | 5·8 | 380 | 1·3 | 30 | 0·6 |
| | | 4 | 6·6 | 300 | 1·5 | 24 | 0·9 |
| | | 11 | 6·3 | 145 | 0·9 | 24 | 5·0 |
| AL | | 14 | 6·2 | 115 | 1·7 | 12·5 | 11·6† |
| IASA | S.A. | — | 8·0 | 350 | 3·0 | 10 | 0·7 |
| IASA | W.A. | — | 7·5 | 180 | 2·8 | 22 | 1·7 |
| IASA | G.M. | — | 10·1 | 175 | 3·2 | 25 | 1·5 |
| | | 9 | 10·2 | 170 | 5·2 | 20 | 1·0 |
| IASA | V.M. | — | 8·7 | 495 | 2·9 | 34 | 1·5 |
| | | 2 | 9·6 | 310 | 3·4 | 12 | 4·0 |
| | | 8 | 9·3 | 290 | 5·3 | 20 | 3·0 |
| SSA | I.L. | — | 11·4 | 260 | 6·8 | 21 | 1·1 |
| SSA | C.K. | — | 8·6 | 145 | 3·0 | 14 | 2·3 |
| SSA | V.F. | — | 12·9 | 150 | 6·5 | 2 | 1·7 |
| HSA | K.P. | — | 12·2 | 290 | 3·7 | 9 | 1·1 |

AL—Acute leukaemia; HSA—hereditary sideroblastic anaemia; IASA—idiopathic acquired sideroblastic anaemia; SSA—secondary sideroblastic anaemia.

* Time represents months from initial presentation.

\+ Plus 26 myeloblasts.

† C.W. and H.S. went on to develop over 50% marrow myeloblasts, and both died of acute leukaemia.

granulopoietic progenitor cells (CFU-C) is based on the linear relationship between colony formation and the number of nucleated cells originally added to the plate. To assay CSA, non-adherent bone marrow cells are obtained after 2 adherence cycles as described by Messner, Till and McCulloch (1973). Such non-adherent cell preparations yield few or no colonies in the absence of added stimulator but a linear increase in colony formation is observed with increasing concentration of CSA. For certain purified preparations, colony formation is inhibited at excess concentrations of CSA (Price, McCulloch and Till, 1973).

*Purification of colony stimulating activity from leucocyte conditioned media*.—Colony stimulating activity was purified from media conditioned by peripheral leucocytes as described previously (Price *et al.*, 1975*a*). When the starting material is media conditioned by normal leucocytes, Sephadex G-150 column filtration of semi-purified CSA discloses 3 apparent peaks of activity, of molecular weights of approximately 90,000, 35,000 and 15,000 (Price *et al.*, 1975*a*). When the starting material is media conditioned by leucocytes from patients with leukaemia in relapse, assays of similar fractions show only one apparent peak of colony stimulating activity (Price *et al.*, 1975*a*).

*Assay for release of virus-like particles in culture*.—The procedure for detecting the release of virus-like particles in cultures of human marrow has been described (Mak *et al.*, 1974). Medium from cultures containing $5 \times 10^6$ nucleated leukaemic marrow cells/ml and supplemented with medium conditioned by leucocytes from a patient with haemochromatosis, is harvested after 5–8 days of incubation. These media and the cells are then assayed for RNA-dependent DNA polymerase (reverse transcriptase) activity associated with particles of densities between 1·22 and 1·17 g/ml as determined by centrifugation on linear sucrose gradients. The reverse transcriptase was assayed by measuring its capacity to catalyse the incorporation of radiolabelled deoxyguanosine triphosphate (dGTP) into DNA either endogenously or when stimulated by the artificial template poly-(rC)$(dG)_{12-18}$ (Baltimore and Smoler, 1971; Scolnick *et al.*, 1972). A sample was considered positive for reverse transcriptase activity when significant stimulation (greater than 1000 ct/min) was observed with the artificial template poly-(rC)$(dG)_{12-18}$ compared with endogenous activity in association with one or more discrete peaks at densities of 1·16–1·23 g/ml in sucrose gradients (for example, see Fig. 1). In some instances the primer $(dG)_{12-18}$ was used as a control for terminal transferase (McCaffery *et al.*, 1975); in these instances, addition of this primer yielded values similar to those of the endogenous reaction.

*Cell separation at unit gravity*.—In some experiments, marrow cells were fractionated by unit gravity sedimentation using the "staput" apparatus originally described by Miller and Phillips (1969). In this procedure, cells sediment through a shallow gradient of foetal calf serum: the method separates populations principally on the basis of cell size.

### RESULTS

### *Granulopoiesis in culture*

Two parameters of granulopoiesis in culture were assessed using cells obtained from patients with sideroblastic anaemia. Granulopoietic colony formation by marrow cells was measured in culture containing media conditioned by leucocytes and known to be an active source of CSA. Conditioned media were prepared from the peripheral leucocytes of patients with sideroblastic anaemia: after purification of CSA, the number of species of molecules with colony stimulating activity produced by these leucocytes was determined. Results of both classes of assay are presented (Table II) together with the subsequent course of the patients. Colony forming capacity of the marrow of the patients was normal or moderately increased (patient CK) in all but one patient. In patient CW, colony formation was below normal limits and this patient subsequently developed acute myelogenous leukaemia (AML). The 3 non-dialysable species of CSA were regularly purified from media prepared from leucocytes of the patients with SSA or HSA and in 3 of the patients with IASA. Leucocytes from 3 of the latter group released only one non-dialysable species of CSA into conditioned media: of these

TABLE II.—*Cell Culture Findings**

| Diagnosis | Patient | CFU-C† | Species of HMW-CSA‡ | Follow-up |
|---|---|---|---|---|
| IASA | C.W. | 15 | 35 | AML§ after 5 months |
| | H.S. | 57 | 35 | AML after 14 months |
| | S.A. | 61 | 35 | Myocardial infarction after 3 months |
| | W.A. | 53 | 15, 35, 90 | Unchanged after 18 months |
| | G.M. | 82 | 15, 35, 90 | Unchanged after 19 months |
| | V.M. | 105 | 15, 35, 90 | Unchanged after 14 months |
| SSA (alcoholism) | I.L. | 83 | 15, 35, 90 | Unchanged after 25 months |
| (chronic pancreatitis) | C.K. | 205 | 15, 35, 90 | Responded to B12 and pancreozymin |
| | V.F. | 82 | 15, 35, 90 | Lymphoma and sideroblastosis persist at 24 months |
| HSA | K.P. | 113 | 15, 35, 90 | Partial response to pyridoxine |

* Data obtained at the time of initial patient evaluation.
† CFU-C, granulocyte colonies per $10^5$ nucleated marrow cells (normal 50–100).
‡ HMW-CSA, prepared from normal leucocytes has species of mol. wt.: $15 \times 10^3$, $35 \times 10^3$ and $90 \times 10^3$ daltons.
§ AML, acute myeloblastic leukaemia.

3 patients, 2 subsequently developed AML and one died 3 months after initial assessment without evidence of leukaemia. The 3 other patients with IASA have remained clinically unchanged for periods ranging from 13 to 19 months. Thus, only one non-dialysable species of CSA was detected in 3 of 6 patients with IASA, a finding regularly obtained on examination of semi-purified CSA from media conditioned by leukaemic leucocytes; further, of these 3 patients, 2 subsequently developed leukaemia.

*Reverse transcriptase activity in supernatants of marrow cultures*

Marrow cells were cultured in a search for evidence of production of virus-like particles, as described in Materials and Methods. Positive results were obtained in only 2 of 10 patients examined: typical findings are shown diagrammatically in Fig. 1. The figure represents profiles of endogenous and primer-stimulated reverse transcriptase activity in sucrose gradients. The top panel of the figure depicts data obtained from one of 5 marrow samples of patient HS, assessed in culture from one to 10 months before the development of AML: all 5 samples from this patient showed similar results. The middle panel depicts the results of one of 2 specimens of marrow from patient VM; reverse transcriptase activity

FIG. 1.—Endogenous and poly-(rC)(dG)$_{12-18}$ stimulated DNA polymerase activity from supernatants of marrow cell cultures of 3 patients with IASA.

After culture, the supernatant from marrow cell cultures of 3 patients with IASA were fractionated and analysed in 20–70% sucrose gradients. Fractions were collected and assayed for endogenous (●——●) and poly-(rC)(dG)$_{12-18}$ stimulated (○——○) DNA polymerase activity.
Top panel: Patient HS
Middle panel: Patient VM
Bottom panel: Patient WA
Background count of 200 ct/min per fraction were substracted. One pmol of $H_3$dGTP is equivalent to 2000 ct/min.

was obtained in low amounts on both marrow specimens: this patient remains clinically unchanged after 13 months of observation. It is evident that the culture supernatants obtained from marrow specimens of each patient contained enzyme activity associated with particles of densities between 1·22 and 1·17 g/ml. In each case, the potential stimulation of incorporation was observed with the artificial template poly-(rC)$(dG)_{12-18}$. The bottom panel depicts the result from patient WA and is representative of results obtained in cultures of marrow from the 8 other patients with IASA, SSA or HSA: similar results have been obtained in normal patients previously reported (Mak *et al.*, 1974).

*Analysis by sedimentation at unit gravity*

The data shown in Fig. 1 indicate the presence of virus-like reverse transcriptase activity in the supernatants of cultures of marrow cells from HS, a patient with IASA whose leucocytes added only a single species of HMW-CSA to culture media, and who later developed AML. In contrast, the marrow cells of VM, a patient with IASA, released virus-like enzyme activity, although her leucocytes were able to add 3 species of HMW-CSA to culture media and she has not developed leukaemia over 13 months' observation. A method of more detailed analysis became available from recent experiments (Mak *et al.*, 1975*b*) in which leukaemic and normal marrow was analysed by velocity sedimentation. In these experiments, poly-(rC)$(dG)_{12-18}$ stimulated reverse transcriptase activity was associated with rapidly sedimenting cells in 6 of 12 marrow specimens from patients with leukaemia, while lesser enzyme activity was found in slowly sedimenting (small) cells in 4 or 11 marrow specimens obtained from patients without leukaemia. The same techniques were applied to marrow from patients HS and VM: for the former patient, examination was made after the appearance of leukaemia (14% myeloblasts in the marrow). The marrow of patient VM was morphologically similar to that obtained at the time of the first examination.

Each specimen was fractionated by velocity sedimentation. Fractions were pooled to yield 4 suspensions, each containing between 3·5 and 5 × $10^7$ cells/ml. These pools were cultured as described in Materials and Methods and after 5 days, cells and culture supernatants were fractionated in sucrose density gradients and fractions examined for the presence of reverse transcriptase activity. Results are shown in Fig. 2 (HS) and

FIG. 2.—Distribution of reverse transcriptase activity in marrow cells from patient HS fractionated by velocity sedimentation. Velocity sedimentation profile of nucleated marrow cells is shown in the lower panel. Cells are combined to yield 4 pools and cultured for 7 days. The amount of reverse transcriptase activity as stimulated by poly-(rC)$(dG)_{12-18}$ in cells (■) and supernatant medium (□) for each pool was assayed as described (see text). Total enzyme activity is illustrated as bar graphs in the upper panel.

FIG. 3.—Distribution of reverse transcriptase activity in supernatant of cultures of marrow cells from patient VM. (For details, see Fig. 2.)

Fig. 3 (VM). In each figure the bottom panel depicts the total cell profile. The top panel indicates the range of sedimentation velocities combined to make each of 4 pools cultured in the assay for the detection of virus-like reverse transcriptase. Each bar in the upper panel represents the ct/min summed over discrete peaks of activity in sucrose density fractions corresponding to densities from 1·17 to 1·22 g/ml. It is clear that for patient VM, poly-(rC)(dG)$_{12-18}$ stimulated reverse transcriptase activity was associated with only slowly sedimenting, small cells. In contrast, patient HS shows activity present both in pools containing small cells and pools containing rapidly sedimenting large cells. The pattern observed in patient VM is similar to that reported for some non-leukaemic marrow specimens, while patient HS shows virus-like reverse transcriptase activity associated not only with small cells but also with large cells, similar to the pattern characteristically shown in marrow from patients with leukaemia (Mak *et al.*, 1975*b*).

### DISCUSSION

The present study shows that peripheral blood and bone marrow cell culture characteristics similar to those occurring in acute leukaemia are also present in sideroblastic anaemia. Correlation between the behaviour of cells from patients with idiopathic acquired sideroblastic anaemia and those from patients with leukaemia was found only in the distribution of non-dialysable species of CSA released into media. Studies of reverse transcriptase activity indicate that caution is required when interpreting such enzymatic data in relation to a patient's risk of developing leukaemia.

Granulopoietic colony formation in culture when applied to the study of human acute leukaemia has yielded variable results (Moore *et al.*, 1974; Curtis *et al.*, 1975), so that the finding of reduced colony formation in one preleukaemic sideroblastic patient (CW, see Table I) and in previous studies of preleukaemia (Greenberg *et al.*, 1971; Senn and Pinkerton, 1972) is not diagnostic of leukaemic change. In acute leukaemia, colony stimulating activity is often altered quantitatively (Messner *et al.*, 1973) and qualitatively (Price *et al.*, 1975*a*, *b*). Three species of non-dialysable CSA can be identified in association with membranes of normal leucocytes (Price *et al.*, 1975*b*) and all these classes are released into culture media. Physically similar species are present in the membranes of leukaemia leucocytes but only one of these 3 non-dialysable species is released in culture (Price *et al.*, 1975*a*). These findings have been interpreted as indicating a reduction in the availability for regulatory interaction of bioactive molecules in leukaemic cells as compared to normal (McCulloch *et*

*al.*, 1974). The present study demonstrates a single species of non-dialysable CSA released into culture media by leucocytes from 3 of 6 patients with IASA; 2 of these patients later developed leukaemia, the third died of myocardial infarction 3 months after the initial assessment of leucocyte function. The detection of this CSA abnormality many months before the development of overt leukaemia suggests that the cultural effect may represent an early granulopoietic evidence of leukaemic transformation.

The particles released by human leukaemic marrow cells in culture have many of the biochemical, physical and morphological characteristics usually associated with leukoviruses (for a recent summary, see McCulloch *et al.*, 1974). In the present study, particle release was assessed only in terms of reverse transcriptase activity associated with appropriate densities in sucrose gradients: this limited criterion was used because the artificial template poly-(rC)$(dG)_{12-18}$ is considered to have specificity for the reverse transcriptases of leukoviruses (Baltimore and Smoler, 1971; Scolnick *et al.*, 1972). Marrow cells from 2 patients with IASA showed enzyme activity with these characteristics. One of these patients (HS) later developed leukaemia, and the other (VM) remains free of clinically evident leukaemia. Recently, the release of small amounts of reverse transcriptase has been associated with non-leukaemic marrow cells of a low sedimentation velocity (Mak *et al.*, 1975*b*). Marrow cells from patient VM produced reverse transcriptase activity in association with slowly sedimenting cells, and thus this activity is similar to that observed in other patients without leukaemia. This experience underlines the need to assess the source of reverse transcriptase activity when it is proposed to use this enzyme as a marker of leukaemic or preleukaemic cells.

This study suggests that cell culture abnormalities, particularly alteration in CSA species released into culture media, may contribute to the evaluation of human populations known to be especially susceptible to leukaemia. The finding of virus-like reverse transcriptase production by the cells of patients reported here, and by Vosika *et al.* (1975) must be confirmed more widely before definite prognostic or aetiological implications are possible. In addition, however, studies such as this support the as yet unproven contention that characteristics displayed by cells in culture are relevant to their behaviour in normal and pathological states *in vivo*.

Supported by grant from the Medical Research Council of Canada (MT1420) and the Ontario Cancer Treatment and Research Foundation (236), and the National Cancer Institute of Canada. We thank Ms J. Grover, K. Benzing, Schachtschabel and M. Bates for capable technical assistance.

### REFERENCES

BALTIMORE, D. & SMOLER, D. (1971) Primer Requirement and Template Specificity of the DNA Polymerase of RNA Tumor Viruses. *Proc. natn. Acad. Sci. U.S.A.*, **68**, 1507.

BJORKMAN, S. E. (1956) Chronic Refractory Anemia with Sideroblastic Bone Marrow. A Study of Four Cases. *Blood*, **11**, 250.

CURTIS, J. E., COWAN, D. H., BERGSAGEL, D. E., HASSELBACK, R. & MCCULLOCH, E. A. (1975) Adult Acute Leukemia: Remission Induction Using Combination Chemotherapy Assessed by Clinical and Cell Culture Criteria. *Can. med. Ass. J.*, **113**, 289.

DAMESHEK, W. (1965) Sideroblastic Anaemia: Is this a Malignancy? *Br. J. Haemat.*, **11**, 52.

GALLAGHER, R. E. & GALLO, R. C. (1975) Type C RNA Tumor Virus Isolated from Cultured Human Acute Myelogenous Leukemia Cells. *Science, N.Y.*, **187**, 350.

GOLDE, D. W. & CLINE, J. J. (1973) Human Preleukemia: Identification of a Maturation Defect *In Vitro*. *N. Engl. J. Med.*, **288**, 1083.

GREENBERG, P. L., NICHOLS, W. C. & SCHRIER, S. L. (1971) Granulopoiesis in Acute Myeloid Leukemia and Preleukemia. *New Engl. J. Med.*, **284**, 1225.

ISCOVE, N. N., SENN, J. S., TILL, J. E. & MCCULLOCH, E. A. (1971) Colony Formation by Normal and Leukemic Human Marrow Cells in Culture: Effect of Conditioned Medium from Human Leukocytes. *Blood*, **37**, 1.

MAK, T. W., MANASTER, J., HOWATSON, A. F., MCCULLOCH, E. A. & TILL, J. E. (1974) Particles with Characteristics of Leukoviruses in Cultures of Marrow Cells from Leukemic Patients in Remission and Relapse. *Proc. natn. Acad. Sci. U.S.A.*, **71**, 4336.

MAK, T. W., KURTZ, S., MANASTER, J. & HOUSMAN, D. (1975*a*) Viral Related Information in Oncornavirus-like Particles Isolated from Cultures of Marrow Cells from Leukemic Patients in Relapse and Remission. *Proc. natn. Acad. Sci. U.S.A.*, **72**, 623.

MAK, T. W., PRICE, G. B., NIHO, Y., MILLER, R. G., SENN, J. S., CURTIS, J. E., TILL, J. E. & MCCULLOCH, E. A. (1975*b*) Enrichment by Velocity Sedimentation of Human Leukemic Marrow Cells Producing Viral-related Particles in Culture. Presented at the Annual Meeting of the American Association for Cancer Research, San Diego, 6 May.

MCCAFFERY, R., HARRISON, T. A., PARKMAN, R. & BALTIMORE, D. (1975) Terminal Deoxynucleotidyl Transferase Activity in Human Leukemic Cells and in Normal Human Thymocytes. *N. Engl. J. Med.*, **292**, 775.

MCCULLOCH, E. A., MAK, T. W., PRICE, G. B. & TILL, J. E. (1974) Organization and Communication in Populations of Normal and Leukemic Hemopoietic Cells. *Biochim., biophys. Acta*, **355**, 260.

MESSNER, H. A., TILL, J. E. & MCCULLOCH, E. A. (1973) Interacting Cell Populations Affecting Granulopoietic Colony Formation by Normal and Leukemic Human Marrow Cells. *Blood*, **42**, 701.

METCALF, D., CHAN, S. H., STANLEY, E. R., MOORE, M. A. S., GUNZ, F. W. & VINCENT, P. C. (1972) Regulation of Normal and Leukaemic Granulocytic Cells by Colony Stimulating Factor (CSF). In *The Nature of Leukaemia*. Ed. P. Vincent. Proc. Int. Cancer Conf., Sydney, Australia, 173.

MILLER, R. G. & PHILLIPS, R. A. (1969) Separation of Cells by Velocity Sedimentation. *J. cell. Physiol.*, **73**, 191.

MOORE, C. V. (1972) Sideroblastic Anemia. In *Hematology*. First Edn. W. J. Williams, E. Beutler, A. J. Erslev and W. R. Rundles. New York: McGraw-Hill. Inc. p. 349.

MOORE, M. A. S., SPITZER, G., WILLIAMS, N., METCALF, D. & BUCKLEY, J. (1974) Culture Studies in 127 Cases of Untreated Acute Leukemia: The Prognostic Value of Reclassification of Leukemia According to *in vitro* Growth Characteristics. *Blood*, **44**, 1.

PRICE, G. B., MCCULLOCH, E. A. & TILL, J. E. (1973) A New Human Low Molecular Weight Granulocyte Colony Stimulating Activity. *Blood*, **42**, 321.

PRICE, G. B., SENN, J. S., MCCULLOCH, E. A. & TILL, J. E. (1975*a*) The Isolation and Properties of Granulocytic Colony Stimulating Activities from Human Peripheral Leucocyte Conditioned Medium. *Biochem. J.*, **148**, 209.

PRICE, G. B., MCCULLOCH, E. A. & TILL, J. E. (1975*b*) Cell Membranes as Sources of Granulocyte Colony Stimulating Activity. *Expl Haemat.*, **3**, 277.

SCOLNICK, E. M., PARKS, W. D., TODARO, G. J. & AARONSON, S. A. (1972) Immunological Characterization of Primate C-Type Virus Reverse Transcriptase. *Nature, New Biol.*, **235**, 35.

SENN, J. S., MCCULLOCH, E. A. & TILL, J. E. (1967) Comparison of the Colony Forming Ability of Normal and Leukaemic Human Marrow in Cell Culture. *Lancet*, ii, 597.

SENN, J. S. & PINKERTON, P. H. (1972) Defective *In Vitro* Colony Formation by Human Bone Marrow Preceding Overt Leukaemia. *Br. J. Haemat.*, **23**, 277.

VOSIKA, G. J., KRIVIT, W., GERRARD, J. M., COCCIA, P. F., NEWBIT, M. E., COALSON, J. J. & KENNEDY, B. J. (1975) Oncornavirus-like Particles from Cultured Bone Marrow Cells Preceding Leukemia and Malignant Histiocytosis. *Proc. natn. Acad. Sci. U.S.A.*, **72**, 2804.